# Pathophysiological Mechanisms Explaining the Association Between Low Skeletal Muscle Mass and Cognitive Function

**DOI:** 10.1093/gerona/glac121

**Published:** 2022-06-06

**Authors:** Susanne Janette Oudbier, Jorming Goh, Stéphanie Marcella Leonie Maria Looijaard, Esmee Mariëlle Reijnierse, Carolus Gerardus Maria Meskers, Andrea Britta Maier

**Affiliations:** Department of Outpatient Clinics, Amsterdam Public Health Research Institute, Amsterdam UMC Location Vrije Universiteit Amsterdam, Amsterdam, The Netherlands; Healthy Longevity Translational Research Program and Department of Physiology, Yong Loo Lin School of Medicine, National University of Singapore, Singapore, Singapore; Centre for Healthy Longevity, @AgeSingapore, National University Health System, Singapore, Singapore; Department of Internal Medicine, Alrijne Hospital, Leiderdorp, The Netherlands; Rehabilitation Medicine, Amsterdam UMC Location Vrije Universiteit Amsterdam, Amsterdam, The Netherlands; Amsterdam Movement Sciences, Ageing and Vitality, Amsterdam, The Netherlands; Department of Medicine and Aged Care, @AgeMelbourne, The Royal Melbourne Hospital, The University of Melbourne, Parkville, Victoria, Australia; Rehabilitation Medicine, Amsterdam UMC Location Vrije Universiteit Amsterdam, Amsterdam, The Netherlands; Amsterdam Movement Sciences, Ageing and Vitality, Amsterdam, The Netherlands; Healthy Longevity Translational Research Program and Department of Physiology, Yong Loo Lin School of Medicine, National University of Singapore, Singapore, Singapore; Centre for Healthy Longevity, @AgeSingapore, National University Health System, Singapore, Singapore; Department of Medicine and Aged Care, @AgeMelbourne, The Royal Melbourne Hospital, The University of Melbourne, Parkville, Victoria, Australia; Department of Human Movement Sciences, @AgeAmsterdam, Faculty of Behavioral and Movement Sciences, VU University Amsterdam, Amsterdam Movement Sciences, Amsterdam, The Netherlands

**Keywords:** Dementia, Inflammation, Insulin, Myokines

## Abstract

Low skeletal muscle mass is associated with cognitive impairment and dementia in older adults. This review describes the possible underlying pathophysiological mechanisms: systemic inflammation, insulin metabolism, protein metabolism, and mitochondrial function. We hypothesize that the central tenet in this pathophysiology is the dysfunctional myokine secretion consequent to minimal physical activity. Myokines, such as fibronectin type III domain containing 5/irisin and cathepsin B, are released by physically active muscle and cross the blood–brain barrier. These myokines upregulate local neurotrophin expression such as brain-derived neurotrophic factor (BDNF) in the brain microenvironment. BDNF exerts anti-inflammatory effects that may be responsible for neuroprotection. Altered myokine secretion due to physical inactivity exacerbates inflammation and impairs muscle glucose metabolism, potentially affecting the transport of insulin across the blood–brain barrier. Our working model also suggests other underlying mechanisms. A negative systemic protein balance, commonly observed in older adults, contributes to low skeletal muscle mass and may also reflect deficient protein metabolism in brain tissues. As a result of age-related loss in skeletal muscle mass, decrease in the abundance of mitochondria and detriments in their function lead to a decrease in tissue oxidative capacity. Dysfunctional mitochondria in skeletal muscle and brain result in the excessive production of reactive oxygen species, which drives tissue oxidative stress and further perpetuates the dysfunction in mitochondria. Both oxidative stress and accumulation of mitochondrial DNA mutations due to aging drive cellular senescence. A targeted approach in the pathophysiology of low muscle mass and cognition could be to restore myokine balance by physical activity.

Diagnostic measures of sarcopenia in older adults, that is, low skeletal muscle mass, muscle strength, and physical performance (1), are associated with detrimental health outcomes and morbidity, that is, falls and fractures (2), caregiver dependence in activities of daily living (3), diabetes (4), and cognitive decline/impairment (5). The prevalence of sarcopenia in older community-dwelling individuals with dementia is more than 3 times higher than individuals without dementia (4). Diagnostic criteria for sarcopenia (eg, low handgrip strength and muscle mass) are independent risk factors for cognitive decline in community-dwelling older individuals (3,6). Despite such strong epidemiological associations, the underlying pathophysiological mechanisms between measures of sarcopenia and cognitive impairment and dementia are yet to be fully established.

A direct link between the skeletal muscle and brain axis has been demonstrated by the release of exercise-induced myokines (7,8). Similarly, insulin metabolism (9), protein metabolism (10), mitochondrial function (11), and systemic inflammation (12) have been suggested to be altered in individuals with low skeletal muscle mass and may contribute to the development of cognitive impairment. Whether these mechanisms drive the pathophysiology of cognitive impairment and therewith explain the association of sarcopenia and cognitive impairment, or whether these mechanisms are indirect links between sarcopenia and cognitive impairment, is debatable. Sarcopenia may be a neurogenic syndrome, as there is a strong link between the central nervous system and muscle via motor neuron connectivity (13). However, in this review, we will focus on the unidirectional relationship between low skeletal muscle mass and cognition. Identification of the underlying pathophysiology may pave the way for novel treatments and targeted interventions to improve the metabolic and functional quality of skeletal muscle mass to reverse sarcopenia, and possibly also improve cognitive function. This narrative review aims to describe the pathophysiological mechanisms of muscle mass loss that may underlie its association with cognitive impairment.

## Literature Search

The literature search for this narrative review comprised 2 databases (PubMed, Embase) and was executed in collaboration with a medical data specialist. Administered indexing terms (eg, MeSH in PubMed and Emtree in Embase) and free-text search terms included (a) muscle mass and strength, (b) cognitive decline and dementia, and (c) predetermined pathophysiological mechanisms: “insulin resistance,” “mitochondrial function,” “protein metabolism,” and “inflammation” (Supplementary Appendix). Reference lists of relevant articles were screened for additional relevant articles.

In our review, we investigated the unidirectional relationship between low skeletal muscle mass and cognitive impairment, and their possible predetermined mechanisms. We do not exclude the possibility that other mechanisms that govern the coupling of skeletal muscle mass and cognitive dysfunction might exist.

## Understanding Skeletal Muscle Mass Loss

Skeletal muscle mass loss occurs during aging and is caused by atrophy of muscle fibers and a decrease in the number of myofibers. After the age of 30 years, muscle mass declines at a rate of approximately 3%–8% per decade and accelerates from 60 years onwards (14). Skeletal muscle fibers are classified into slow-twitch type I and fast-twitch type II fibers. Type II fibers predominate high glycolytic capacity, whereas type I fibers comprise a higher mitochondrial content and oxidative capacity (15). During skeletal muscle aging, the loss of type II fibers occurs prior to the loss of type I fibers (16).

The decline of immune function caused by aging is characterized by a systemic increase in proinflammatory cytokines, such as interleukin (IL)-1β, IL-6, and tumor necrosis factor-α (TNF-α) (16), resulting in a chronic low inflammatory state. Systemic inflammation and thereby uncontrolled release of inflammatory cytokines result in dysfunction of mitochondria, with a negative spiral in which less energy (ATP) is produced, and reactive oxygen species (ROS) further exacerbates damage to mitochondria (16). In addition, damaged mitochondria lead to metabolic abnormalities such as insulin resistance and the activation of the ubiquitin–proteasome system (UPS), which plays a major role in muscle degradation (17).

Fundamental to muscle fiber atrophy is the occurrence of anabolic resistance, which is the suppression of protein synthesis to anabolic stimuli, such as an increased amino acid availability (16). As a typical response to amino acid availability, signaling pathways for synthesis will be activated by mammalian target of rapamycin (mTOR). However, mTOR activation is reduced in older individuals (18). Given isocaloric amounts of protein, protein synthesis is also reduced in older individuals (16).

As a result of aging, the number of mitochondria is reduced with an increased number of mitochondrial DNA (mtDNA) mutations, which may drive apoptosis (19). Moreover, oxidative stress drives oxidative damage, as the production of ROS increases (19). ROS further exacerbates damage to mitochondria, with a vicious cycle in which dysfunctional mitochondria beget dysfunctional mitochondria (19).

## Understanding Cognitive Dysfunction

Aging is the largest risk factor for loss of muscle mass and cognitive decline. The World Health Organization (WHO) estimated that 55 million individuals worldwide are living with dementia (20). As the number of old adults increases, it is predicted that the number of individuals with dementia will double every 5 years (21).

Age-related changes that correlate with cognitive decline include loss of synapses, dysfunction in neuronal networks, and changes in neuronal structure without neuronal death (22). Neuronal loss that occurs with aging in the central nervous system (CNS) is limited (<10%) and restricted to certain regions (23). The changes that occur to neurons are, for instance, a decrease in the number of axons, loss of synapses, decrease in number and length of dendrites, and a loss of dendritic spines (22). It is suggested that these morphological changes contribute significantly to cognitive decline (22,23). The purported molecular mechanisms that connect loss of muscle mass with cognitive decline include altered myokine secretion, inflammation, insulin resistance, abnormal protein accumulation, oxidative stress, and mitochondrial dysfunction (24).

Neuroinflammation may play a role in cognitive decline. An increase in peripheral proinflammatory cytokines during aging can exert its effects in the brain by directly crossing the blood–brain barrier (BBB), and activate microglia, resulting in neuroinflammation in the CNS (24). In Alzheimer’s disease (AD), the role of neuroinflammation is clearer because Aβ contributes directly to microglial activation (24). Microglia are resident macrophages in the brain, which mediate this inflammatory response. However, it is suggested that altered morphology of microglia leads to altered functions that enhance the production of proinflammatory cytokines (24).

Insulin in the brain is mainly originated from insulin secreted by β cells in the pancreas and transported via a saturable receptor-mediated pathway (25). Insulin becomes active as a tyrosine kinase when bound to its receptor (26). The number of insulin receptors in the brain reduces with aging (26). Aberrant insulin uptake makes the brain susceptible to neurodegeneration and cognitive dysfunction, as it is important in neuroprotection (25). Underlying mechanisms of insulin resistance leading to cognitive dysfunction are an increased tau protein concentration, altered hippocampal plasticity, altered amyloid precursor protein (APP) metabolism, and altered inflammatory response in the brain (25,27).

With regard to protein metabolism, mTOR is integral in nutrient sensing and protein synthesis, regulating cellular proliferation, growth, and senescence (18). Aging is linked to alterations in protein synthesis, degradation, folding, and trafficking. To maintain proteostasis, protein clearance (via autophagy or proteosomal degradation) and protein synthesis are important mechanisms influenced by mTOR. In addition, mTOR may underlie mitochondrial dysfunction as it regulates mitochondrial biogenesis, dynamics, and function (18). Therefore, disturbance of mTOR signaling as a result of aging affects mitochondrial function, glucose metabolism, energy production, and autophagy in the brain (28).

Finally, mitochondrial dysfunction occurs as a result of accumulation of mtDNA mutations due to an increase in ROS-mediated molecular damage. Both inflammation and mitochondrial dysfunction increase the production of ROS (29). As a result, impaired mitochondrial function in the brain may lead to a vicious cycle in which an increase of ROS may directly damage mitochondrial proteins and further impair cellular energy production (24,29).

## Overview of Pathophysiological Mechanisms

**Figure 1.**
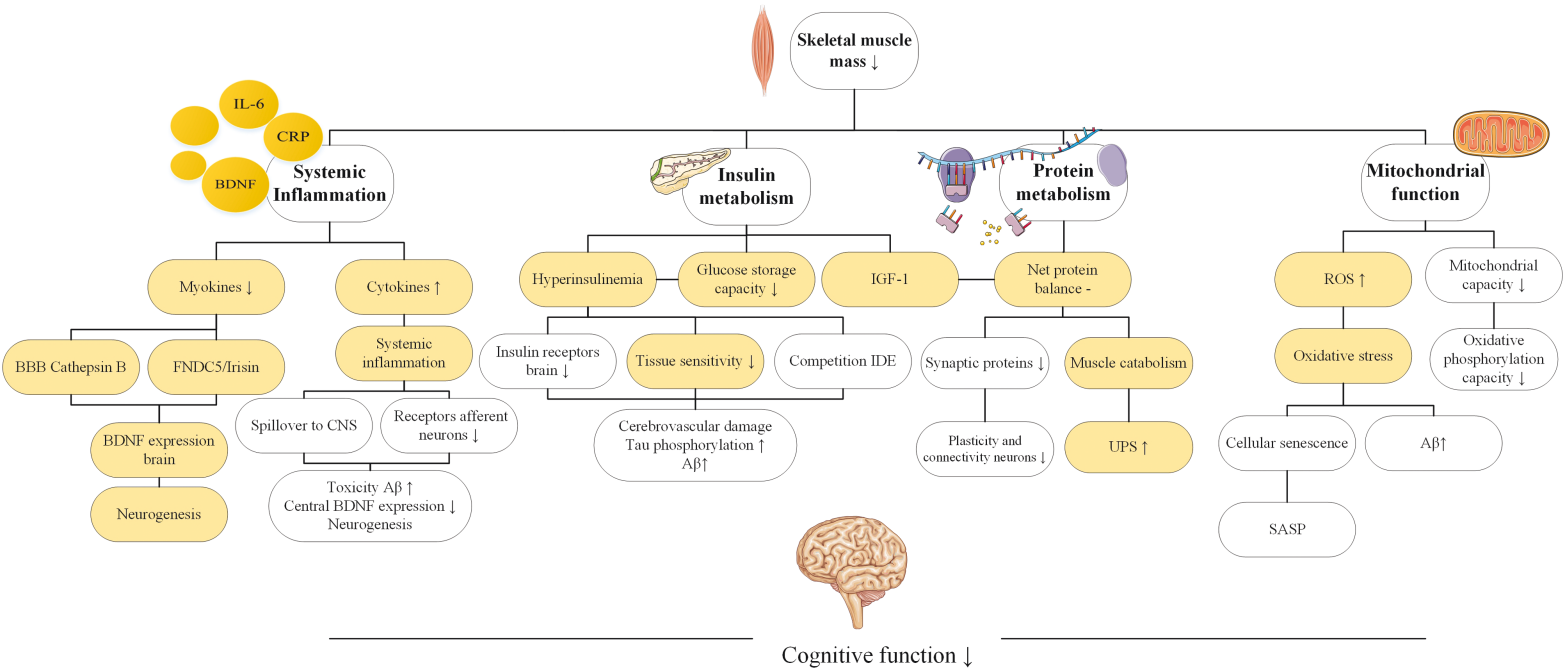
Overview of the purported pathophysiological mechanisms explaining the association between sarcopenia and cognitive function. Yellow marked fields could also be affected by the mediating role of exercise. Aβ = amyloid beta; BBB = blood–brain barrier; BDNF = brain-derived neurotrophic factor; CRP = C-reactive protein; CNS = central nervous system; CDLK-5 = cyclin-dependent-like kinase 5; FNDC5 = fibronectin type III domain containing 5; IDE = insulin-degrading enzyme; IGF-1 = insulin-like growth factor 1; IL-6 = interleukin 6; ROS = reactive oxygen species; SASP = senescence-associated secretory phenotype; UPS = ubiquitin–proteasome system.

Figure 1 gives an overview of the possible pathophysiological mechanisms explaining the association between sarcopenia and cognitive impairment.

### Purported Mechanism 1: Systemic Inflammation

Low skeletal muscle mass in older adults is associated with low-grade, systemic inflammation (30), which in turn is associated with cognitive impairment and dementia (31). First, age-associated physiological changes in the innate and adaptive immune function are called “immunosenescence” (32). Typically, these alterations lead to reduced immune competence to cope with infections, resulting in a compromised adaptive immune system. Second, nonresolving inflammation is also observed (32), which evolves into a low-grade, chronic inflammatory state and has been termed “inflamm-aging” (32). Moreover, systemic concentrations of proinflammatory cytokines originating from adipose tissue are higher in sedentary older individuals, who often have impaired muscle strength and muscle power (33) compared with physically active individuals (34). Similarly, the majority of circulating IL-6 diabetic people originate from adipose tissue (adipocytes and adipose tissue macrophages) (35). Comparatively, healthy nondiabetic individuals have significantly lower concentrations of circulating IL-6, compared with individuals with type 2 diabetes (36). Elevated concentrations of inflammatory markers in the systemic circulation, such as C-reactive protein (CRP) and IL-6, are associated with the risk of dementia (37), although local inflammation in the CNS and systemic inflammation are suggested to contribute to cognitive decline (38). Although the debate on the central or peripheral drivers of cognitive decline and dementia rages on, it is evident that the immune bidirectional crosstalk in the brain–periphery axis is a considerable contributor to this pathology. A purported mechanism is that peripheral proinflammatory cytokines interact via receptors on afferent neurons within the CNS (39). Systemic elevation of proinflammatory cytokines and chemokines can disrupt neurogenesis—plasma C-C motif chemokine ligand 11 (CCL11) demonstrated an age-associated increase in its concentration in plasma and cerebrospinal fluid (CSF) of healthy human volunteers between 20 and 90 years of age (40). In preclinical animal studies, intraperitoneal administration of CCL11 in young mice led to significant decreases in neurogenesis, but this deficit was rescued by systemic administration of CCL11 antibody (40). Furthermore, and more importantly, peripherally derived inflammatory cytokines can augment the CNS-localized inflammatory response, such as leading to the priming, and activation of resident microglial cells, that further drive the production of cytokines (eg, IL-1β, IL-6, and TNF-α) locally (41,42). The long-term activation of microglia and their chronic production of these cytokines can then disrupt normal neural processes, such as disrupting long-term potentiation (43), stimulate the production of, and increase the cytotoxicity of Aβ (44), with the impaired clearance of such neurotoxins further driving the loss of other neuronal integrity.

#### Role of proinflammatory cytokines

The prototypical cytokine, IL-6, behaves as an anti-inflammatory cytokine when secreted by type I and II skeletal muscle fibers during muscle contraction (45). IL-6 plays an important role in the homeostasis of the CNS as it acts with 2 types of signaling in nervous tissue: either via binding to a membrane receptor (classical signaling) or via the soluble form of its receptor (sIL-6R; trans-signaling), generated through alternative mRNA splicing, proteolysis of the membrane-bound IL-6R or through the release of extracellular vesicles (46). Because membrane-bound IL-6R are expressed on hepatocytes, most immune cells, myocytes, and hepatocytes, IL-6 signaling in these cells occurs *via* the classical route (46). Comparatively, trans-signaling occurs in other cell types lacking the membrane-bound IL-6R, such as neurons, where the ubiquitously expressed gp130 membrane protein permits the docking of the IL-6/sIL-6R, becomes a homodimer and leads to intracellular signaling pathways. IL-6 trans-signaling has also been discovered to be the main mechanism through which this cytokine’s proinflammatory functions are mediated (47). In a number of murine models of human inflammatory diseases such as liver cancer, atherosclerosis, inflammatory bowel disease, the blockage of trans-signaling was sufficient to prevent disease progression (48), suggesting that IL-6 trans-signaling is responsible for the relevant pathology, and also during the inflammation seen in CNS aging (49).

Given that obese individuals present with higher plasma concentrations of IL-6R and A disintegrin and metalloprotease 17 (ADAM17), the latter a protease responsible for cleaving membrane-bound IL-6R into its soluble form (50), our working model posits that a combination of inactive muscle and excess adipose tissue (due to chronic sedentary behavior) drives IL-6 trans-signaling during “inflamm-aging” and neurodegeneration. IL-6 released from excess or inflamed adipose tissue bind to soluble IL-6R and mediate detrimental signaling in the CNS (51).

#### Role of myokines

Contracting skeletal muscle behaves like an endocrine organ secreting cytokines and peptides, called “myokines” (52). Muscle atrophy and the loss of type II fibers, and subsequent switch to type I fibers may result in altered secretions of myokines. Myokines can be both pro- and anti-inflammatory and include IL-6, IL-8, IL-15 (53), and brain-derived neurotrophic factor (BDNF) (54), which is summarized elsewhere (52). According to the “Myokine Concept,” physically inactive muscles suppress the endocrine function of muscle, favoring inflammation and thereby increasing the risk of dementia (55). In a 10-year longitudinal study, higher systemic concentrations of IL-6 effectively predicted cognitive decline in late midlife (56). Whether myokines cross the BBB remains speculative, although the fact that cytokines can cross the BBB via circumventricular organs that are in close proximity with the hypothalamus (57) suggest that this mechanism may also apply to myokines. In this respect, recent findings implicate a role for other novel, exercise-induced myokines, such as cathepsin B and FNDC5/irisin (58,59). Cathepsin B crosses the BBB and upregulates local neurotrophin expression, the most well-known and studied being BDNF (58). Exercise increases the gene/protein expression of peroxisome proliferator-activated receptor-gamma coactivator-1-alpha (PGC1α) in skeletal muscle cells, a mediator of oxidative phosphorylation and mitochondrial biogenesis (58). As a result, PGC1α induces the expression of a transmembrane precursor FNDC5, which is then cleaved and releases a peptide, irisin, into the systemic circulation (58). The purported mechanism is that the released myokine irisin crosses the BBB and upregulates the expression of BDNF, thereby influencing neurogenesis and positively affecting memory. However, whether the effect of irisin in the brain is due to exercise (and which type of exercise) is controversial and needs further investigation (58).

In community-dwelling older women, low plasma BDNF concentrations were associated with lower cognitive function (60) and with higher mortality risk during 5 years of follow-up (61). In patients with AD peripheral concentrations of BDNF were significantly lower compared with individuals with no AD (62). Peripheral concentrations of BDNF are of interest because muscular contraction leads to increased BDNF mRNA and protein expression in muscle tissue and an increase in BDNF protein concentrations in the systemic circulation (58). Central BDNF expression and function is reduced by elevated concentrations of proinflammatory cytokines, which affects neurogenesis (63). However, most BDNF found in systemic circulation after exercise was determined to have originated from the brain (52), and there is likely a BDNF threshold that needs to be achieved to modulate neurogenesis (64).

Another possible mechanism is that peripheral irisin concentrations might affect local concentrations of irisin in the brain (65). For instance, in AD models, the effects of exercise on memory and synaptic plasticity were attenuated by blockade of irisin (peripheral and brain) (65). In patients with AD, levels of FNDC5/irisin are lower in hippocampi and CSF compared with healthy individuals or patients with mild cognitive impairment (65). Moreover, a positive association was found between CSF concentrations of irisin and BDNF (66).

### Purported Mechanism 2: Insulin Metabolism

Skeletal muscle plays an important role in glucose homeostasis because it is the dominant tissue responsible for glucose storage and metabolism (67). Low skeletal muscle mass is associated with insulin resistance (68), and insulin resistance is an independent risk factor for cognitive decline (69). Impaired glucose tolerance often accompanies insulin resistance with aging (70). In older individuals without cognitive dysfunction, impaired glucose tolerance with aging was associated with longitudinal changes in brain function (regional cerebral blood flow) (71). Because energy metabolism in brain tissue is mainly dependent on glucose (72), elevated glucose concentrations in peripheral tissues impede normal glucose regulation and insulin sensitivity in the brain (73).

Insulin resistance increases with age (9). Both glucose and insulin pass the BBB and have intracerebral effects, such as synaptic remodeling, regulating the expression of neurotransmitters, and acting selectively in brain regions to increase glucose metabolism (74). Hyperinsulinemia downregulates the number of insulin receptors in the BBB and, thus, attenuates insulin transport in the brain (74). Eventually, chronic hyperinsulinemia dampens tissue sensitivity to insulin, leading to cerebrovascular damage (74).

Long-term exposure to elevated concentrations of glucose results in inappropriate secretion of insulin leading to hyperinsulinemia, which negatively affects neurons (75). Both utilization and uptake of glucose are impaired in AD (75). Peripheral insulin resistance leading to hyperinsulinemia affects insulin signaling in the CNS, which stimulates tau phosphorylation, oxidative stress, and toxicity of Aβ, contributing to cognitive decline (75).

The transport of insulin across the BBB occurs primarily via an energy-dependent saturable transport system (76). When insulin reaches the brain, it can bind to microvessels. Insulin binding to brain microvessels was higher in a mouse model of AD (aged mice compared with young mice). However, whether this is related to altered insulin levels at the transporter level is unclear (76). Thus, it has been described that chronic elevation of peripheral concentrations of insulin are associated with relatively lower insulin concentrations in the brain (74). Low insulin concentrations in the brain reduce Aβ clearance due to decreased transport activity of Aβ from intracellular to extracellular compartments, which is considered the primary site for Aβ clearance (74). However, it is unclear whether this can be attributed to the high peripheral insulin concentrations that arise as a consequence of low skeletal muscle mass.

Another suggested mechanism in the link between peripheral hyperinsulinemia and AD is the accumulation of Aβ due to direct competition of insulin-degrading enzyme (IDE) through which Aβ degradation is attenuated, thereby increasing tau formation (75). However, neuronal uptake of glucose is not fully dependent on insulin and therefore, insulin signaling pathways are more ascribed to insulin resistance in the brain (75).

Insulin-growth factor 1 (IGF-1) is intimately involved in protein biosynthesis, and thus, the net balance of protein synthesis and breakdown determines the degree of muscle mass accrual (77). Recent studies found an association between lower serum IGF-1 levels and sarcopenia in older adults (78,79). The bioavailability of peripheral IGF-1 depends on its insulin-like growth factor-binding proteins (IGFBP), of which approximately 80% is bound to IGFBP-3 in the periphery (80). The associations between peripheral IGF-1 and IGFBP-3 have been evaluated, which showed that these measures were associated with cognition in older women, whereas the direction of this association remains to be elucidated (80).

### Purported Mechanism 3: Protein Metabolism

Skeletal muscle mass is negatively affected by decreased muscle protein synthesis (MPS) and increased muscle protein breakdown (MPB), which results in a negative net protein balance (81). Decreased skeletal MPS that accompanies aging is called “anabolic resistance” (82). The age-related decrease in protein synthesis comprises changes in protein folding, maintenance, and breakdown (83). Low muscle mass consequent to a negative net protein balance could also reflect lower protein concentrations in the brain, indirectly affecting cognition. Abnormal depositions of misfolded and aggregated proteins are common in several types of dementia (84,85). Due to oxidative damage, several proteins as markers of oxidative stress accumulate. As revealed by proteomic techniques in postmortem brains of older humans, oxidative damage by lipoxidation with aging occurs mostly in proteins involved in neurotransmission, proteostasis, and energy metabolism (86). These modifications and alterations in proteins are of interest because the degree of oxidative damage to specific proteins is more severe in AD brains (24).

Another probable mechanism linking low skeletal muscle mass with cognition is that low skeletal muscle mass is related to the upregulation of the ubiquitin-dependent proteolytic system (UPS), a major pathway that clears short-lived, damaged, and misfolded nuclear and cytoplasmic proteins and is upregulated in AD (87). The UPS is related to the degradation of proteins and plays an essential role in neuronal signaling such as synaptic activity and neurotransmitter release (87). A key determinant in the AD pathophysiology is APP, an acute-phase protein that is described to be connected with the UPS (87). The suggested pathway is that dysfunction or overload of the UPS may cause accumulation of Aβ in AD (87). In addition, an accumulation of ubiquitin is found in the Aβ deposits and neurofibrillary tangles of AD (87). Although the upregulation of the UPS system has been described in regards to aging and cachexia, the relationship with sarcopenia requires further investigation (88).

### Purported Mechanism 4: Mitochondrial Function

The energetic needs for skeletal muscle contraction are provided by ATP, which is mainly driven by mitochondrial oxidative phosphorylation (89). Skeletal muscle mitochondria fulfill different roles with regard to metabolic regulation, that is, apoptosis, synthesis, and catabolism of metabolites, and production and quenching of ROS (89). Skeletal muscles use oxygen and in turn, produce large amounts of reactive oxygen and nitrogen species (RONS) (90). Under normal conditions, ROS are molecular signal transducers (17); however, overproduction of ROS in dysfunctional mitochondria can lead to increased oxidative stress and damage to organelles (90). One common pathogenic mechanism of both sarcopenia and dementia is the involvement of oxidative stress, which is described as the imbalance between the generation of, and detoxification of RONS in cells (90). One potential pathophysiological mechanism caused by impaired mitochondrial function is called “the oxidative stress theory,” according to which accumulation of RONS possibly leads to cellular senescence (90). Mitochondrial abnormalities (content, function, morphology) are common in individuals with low muscle mass (17). Mitochondrial dysfunction in the brain is also a potentially underlying mechanism in dementia (91,92). Whether peripheral mitochondrial dysfunction affects mitochondria in the brain needs to be clarified. With aging, skeletal muscles oxidative phosphorylation capacity decreases while deletions accumulate in mtDNA (19,93). These mutations can result in oxidative tissue damage (19). Skeletal myocytes are reliant on efficient production of ATP from functional mitochondria, and disruption of this machinery can drive cellular apoptosis—a precursor to loss in muscle tissue (94). Mitochondrial dysfunction drives cellular senescence (95), which blocks cell proliferation and leads to a senescence-associated secretory phenotype (95). This involves a constellation of proinflammatory cytokines and enzymes such as matrix metalloproteases (MMPs), which are a large family of cleavage proteins. MMPs might be a therapeutic target because it can degrade Aβ, and MMP concentration is higher in AD brains (96). However, MMPs can also induce the transcription of inflammatory players (90,97). This may result in a feedback loop wherein oxidative stress and overproduction of ROS contribute to mitochondrial dysfunction (98). In particular, overproduction of ROS increases oxidative stress, which increases secretion and production of Aβ (99,100).

The trafficking and accumulation of Aβ in mitochondria of neurons is associated with impaired mitochondrial function (101). The “mitochondrial cascade hypothesis” suggests that age-associated mitochondrial decline occurs at a certain threshold and is concomitant with synaptic loss, Aβ production, tau phosphorylation, and plaque deposition in the brain (102). With regard to Aβ accumulation, it is suggested that in the first-place neurons will compensate bioenergetically for the decline in mitochondrial mass (103). However, as Aβ production increases, neurons can no longer compensate for the ongoing processes of Aβ accumulation (103). Lastly, a decrease in mitochondrial mass that accompanies the hypometabolic state will decrease Aβ production (103). Clinically, the “mitochondrial cascade” hypothesis suggests that amyloid accumulation already reaches a plateau before the manifestation of the actual symptoms of AD (104)

## Interplay Between Inflammation, Insulin, Protein Metabolism, and Mitochondrial Function; Myokines: Muscle-Derived Peptides Orchestrating Interorgan Crosstalk

Although the pathophysiological mechanisms were described as separate entities, there is an interplay between these mechanisms. We hypothesize that altered myokine release from skeletal muscle are the key modulators of the 4 physiological hallmarks that accompany cognitive decline. Myokines crosstalk with other molecular players in the brain to exert positive effects on neurogenesis, nervous system development, and neuroprotection in response to exercise (7). For instance, FNDC5 enhances neurogenesis by inducing BDNF locally in the hippocampus (105). In addition, the cleaved peptide product of FNDC5, irisin, enhances neuronal proliferation and differentiation. Irisin further stimulates neuroprotection via seronine/threonine kinase AKT/extracellular signal-regulated kinases 1 and 2 signaling pathways (7). In preclinical animal models, 2 weeks of voluntary wheel running resulted in an increase of IL-6 expression in murine hippocampus and a downregulation of the systemic concentrations of proinflammatory cytokines and inflammation (106).

BDNF exerts its beneficial effect on mitochondria by activating AMP-activated protein kinase and enhancing fatty acid oxidation (107). BDNF also upregulates the expression of PGC-1α, a master transcription factor that regulates mitochondrial biogenesis, in cultured hippocampal neurons, thus, illustrating its important role for central energy metabolism (108). PGC-1α is essential for energy metabolism including mitochondrial biogenesis and release of myokines such as FDNC5/Irisin and cathepsin B (107). PGC-1α expression in response to exercise drives secretion of myokines (7). Therefore, physical inactivity or a sedentary lifestyle result in reduced release of myokines, but also contributes to production of proinflammatory cytokines as PGC-1α decreases the activity of NFκB, which regulates proinflammatory gene expression (109).

Cathepsin B regulates the release of proapoptotic molecules and therefore influences mitochondrial cell death signaling (110). An important age-related alteration of the neuromuscular system is the decrease in the number of motor units, which includes the muscle fibers innervated from the peripheral axon (111). The quantity of myokines may be reduced due to low levels of physical activity that could contribute to the loss of motor units that accompanies aging, thereby influencing the aforementioned pathways of interest (inflammation, insulin metabolism, and mitochondria) in the brain microenvironment, via alterations to their paracrine and endocrine signaling (8). In this regard, neurturin, a glial cell line-derived neurotrophic factor, was recently shown to also function as a novel myokine. Neurturin is transcriptionally upregulated in skeletal muscle after voluntary wheel running in mice and after high-intensity sprint cycling in human subjects, and is involved in remodeling the postsynaptic properties of the neuromuscular junction (112). Note that the abovementioned examples do only refer to the interplay of myokines with the other mechanisms and do not comprehensively reflect other possible complex interplays between all the described pathophysiological mechanisms.

## Bidirectional Relationship: Brain and Muscle?

Most of the studies in this review are cross-sectional studies. Longitudinal studies evaluating the pathophysiological mechanisms between muscle mass, and cognition as an outcome are scarce. The described pathophysiological mechanisms are not necessarily unidirectional and could be bidirectional, as cognition could influence skeletal muscle mass in a retrograde manner because similar associations were found between low muscle mass, muscle strength, and cognitive impairment and vice versa (113–115). The key take away from this review is that the described mechanisms may lead to a negative spiral, in which cognitive impairment may further exacerbate the loss in muscle mass and vice versa, and therefore, reverse causation cannot be excluded.

### Future Perspectives

Physical inactivity is a major risk factor for both sarcopenia and cognitive impairment, and most studies highlight the positive effects of physical activity on dementia and cognitive impairment (116,117). In general, physical exercise has a positive influence on cognitive function by increasing synaptic plasticity and the underlying systems that support neurogenesis (104). Various types of exercise affect different pathophysiological pathways; however, this is beyond the scope of this review. As shown by numerous observational studies, there is an inverse association between exposure to nonsteroidal anti-inflammatory drugs and risk of AD, whose effects may vary with the degree of cognitive decline (118,119). Clinical implications regarding myokines are that they cross the BBB and therefore may be a target for therapy as they influence the brain microenvironment. For instance, skeletal muscle produces irisin through cleavage of FNDC5 and then bound to its receptors on neurons, which stimulates expression of BDNF, enhancing cognition (65,120). In addition, the production of FNDC5 in the brain will be stimulated by exercise through PCG-1α (in skeletal muscle), which also stimulates BDNF expression (105). Thus, exercise stimulates BDNF expression in the brain and preserves cognition via irisin-induced signaling mechanisms (120). Recognizing the underlying pathophysiological mechanisms of muscle mass with cognition is important to gain insight into dementia as well as into the development of targeted interventions. Therefore, future research should focus on longitudinal and interventional studies to evaluate the causality of the relationship between muscle mass and cognitive impairment and the interplay of the underlying mechanisms.

## Conclusion

There is substantial evidence supporting 4 pathophysiological mechanisms that may underlie the association between low muscle mass and cognitive impairment, that is, systemic inflammation, insulin, protein metabolism, and mitochondrial function. Low skeletal muscle mass and alterations in myokine secretion leading to inflammation and lower peripheral glucose storage due to low muscle mass are the mechanisms with the most evidence in the association with impaired cognition. However, it remains unclear if these mechanisms are directly or indirectly caused by skeletal muscle mass loss or if a bidirectional relationship exists. The underlying mechanisms of protein metabolism and mitochondrial function because of low skeletal muscle mass needs further exploration in relation to cognition.

## Supplementary Material

glac121_suppl_Supplementary_Appendix

## References

[1] Cruz-Jentoft AJ , Bahat G, Bauer J, et al. Sarcopenia: revised European consensus on definition and diagnosis. Age Ageing. 2019;48(1):16–31. doi:10.1093/ageing/afy169 30312372 PMC6322506

[2] Yeung SS , Reijnierse EM, Pham VK, et al. Sarcopenia and its association with falls and fractures in older adults: a systematic review and meta‐analysis. J Cachexia Sarcopenia Muscle. 2019;10(3):485–500. doi:10.1002/jcsm.12411 30993881 PMC6596401

[3] Wang DX , Yao J, Zirek Y, Reijnierse EM, Maier AB. Muscle mass, strength, and physical performance predicting activities of daily living: a meta‐analysis. J Cachexia Sarcopenia Muscle. 2020;11(1):3–25. doi:10.1002/jcsm.12502 31788969 PMC7015244

[4] Pacifico J , Geerlings MA, Reijnierse EM, Phassouliotis C, Lim WK, Maier AB. Prevalence of sarcopenia as a comorbid disease: a systematic review and meta-analysis. Exp Gerontol. 2020;131:1–19. doi:10.1016/j.exger.2019.110801 31887347

[5] Cipolli GC , YassudaMS, AprahamianI. Sarcopenia is associated with cognitive impairment in older adults: a systematic review and meta-analysis. J Nutr Health Aging.2019;23(6):525–531. doi:10.1007/s12603-019-1188-831233073

[6] Beeri MS , Leugrans SE, Delbono O, Bennett DA, Buchman AS. Sarcopenia is associated with incident Alzheimer’s dementia, mild cognitive impairment, and cognitive decline. J Am Geriatr Soc. 2021;182:6–1835. doi:10.1111/jgs.17206 PMC8286176 33954985

[7] Chen W , Wang L, You W, Shan T. Myokines mediate the cross talk between skeletal muscle and other organs. J Cell Physiol. 2021;239:3–2412. doi:10.1002/jcp.30033 32885426

[8] Severinsen MCK , Pedersen BK. Muscle–organ crosstalk: the emerging roles of myokines. Endocr Rev. 2020;41(4):594–609. doi:10.1210/endrev/bnaa016 PMC7288608 32393961

[9] Cleasby ME , Jamieson PM, Atherton PJ. Insulin resistance and sarcopenia: mechanistic links between common co-morbidities. J Endocrinol. 2016;229(2):R67–R81. doi:10.1530/JOE-15-0533 26931135

[10] Wu H , Jang J, Dridi S, et al. Net protein balance correlates with expression of autophagy, mitochondrial biogenesis, and fat metabolism‐related genes in skeletal muscle from older adults. Physiol Rep. 2020;8(19):e14575. doi:10.14814/phy2.14575 33063954 PMC7556313

[11] Ferri E , Marzetti E, Calvani R, Picca A, Cesari M, Arosio B. Role of age-related mitochondrial dysfunction in sarcopenia. Int J Mol Sci. 2020;21(15):5236. doi:10.3390/ijms21155236 PMC7432902 32718064

[12] Tuttle CSL , Thang LAN, Maier AB. Markers of inflammation and their association with muscle strength and mass: a systematic review and meta-analysis. Ageing Res Rev. 2020;64:101185. doi:10.1016/j.arr.2020.101185 32992047

[13] Kwan P . Sarcopenia, a neurogenic syndrome? J Aging Res. 2013;2013:791679. doi:10.1155/2013/791679 23577254 PMC3610356

[14] Volpi E , Nazemi R, Fujita S. Muscle tissue changes with aging. Curr Opin Clin Nutr Metab Care. 2004;7(4):405–410. doi:10.1097/01.mco.0000134362.76653.b2 15192443 PMC2804956

[15] Yakabe M , Ogawa S, Akishita M. Clinical manifestations and pathophysiology of sarcopenia. RNA Transcr. 2015;1(2):10–17. doi:10.11648/j.rnat.20150102.11

[16] Nishikawa H , Fukunishi S, Asai A, Yokohama K, Nishiguchi S, Higuchi K. Pathophysiology and mechanisms of primary sarcopenia. Int J Mol Med. 2021;48(2):1–8. doi:10.3892/ijmm.2021.4989 34184088

[17] Picca A , Calvani R, Bossola M, et al. Update on mitochondria and muscle aging: all wrong roads lead to sarcopenia. Biol Chem. 2018;399(5):421–436. doi:10.1515/hsz-2017-0331 29384724

[18] Papadopoli D , Boulay K, Kazak L, et al. mTOR as a central regulator of lifespan and aging. F1000Res. 2019;8:9981–9921. doi:10.12688/f1000research.17196.1 PMC6611156 31316753

[19] Peterson CM , Johannsen DL, Ravussin E. Skeletal muscle mitochondria and aging: a review. J Aging Res. 2012;2012. doi:10.1155/2012/194821 PMC3408651 22888430

[20] Fact Sheet Dementia. World Health Organization. https://www.who.int/news-room/fact-sheets/detail/dementia. Updated September 2, 2021. Accessed February 5, 2022.

[21] Cao Q , Tan C-C, Xu W, et al. The prevalence of dementia: a systematic review and meta-analysis. J Alzheimers Dis. 2020;73(3):1157–1166. doi:10.3233/JAD-191092 31884487

[22] Murman DL . The impact of age on cognition. Semin Hear. 2015;36(3):111–121. doi:10.1055/s-0035-1555115 27516712 PMC4906299

[23] Pannese E . Morphological changes in nerve cells during normal aging. Brain Struct Funct. 2011;216(2):85–89. doi:10.1007/s00429-011-0308-y 21431333

[24] Poddar J , Pradhan M, Ganguly G, Chakrabarti S. Biochemical deficits and cognitive decline in brain aging: Intervention by dietary supplements. J Chem Neuroanat. 2019;95:70–80. doi:10.1016/j.jchemneu.2018.04.002 29678666

[25] Ma L , Wang J, Li Y. Insulin resistance and cognitive dysfunction. Clin Chim Acta. 2015;444:18–23. doi:10.1016/j.cca.2015.01.027 25661087

[26] Kullmann S , Heni M, Hallschmid M, Fritsche A, Preissl H, Häring H-U. Brain insulin resistance at the crossroads of metabolic and cognitive disorders in humans. Physiol Rev. 2016;96(4):1169–1209. doi:10.1152/physrev.00032.2015 27489306

[27] Biessels GJ , Reagan LP. Hippocampal insulin resistance and cognitive dysfunction. Nat Rev Neurosci. 2015;16(11):660–671. doi:10.1038/nrn4019 26462756

[28] Perluigi M , Di Domenico F, Butterfield DA. mTOR signaling in aging and neurodegeneration: at the crossroad between metabolism dysfunction and impairment of autophagy. Neurobiol Dis. 2015;84:39–49. doi:10.1016/j.nbd.2015.03.014 25796566

[29] Chakrabarti S , Munshi S, Banerjee K, Thakurta IG, Sinha M, Bagh MB. Mitochondrial dysfunction during brain aging: role of oxidative stress and modulation by antioxidant supplementation. Aging Dis. 2011;2(3):242–256. 22396876 PMC3295058

[30] Tuttle CS , Waaijer ME, Slee‐Valentijn MS, Stijnen T, Westendorp R, Maier AB. Cellular senescence and chronological age in various human tissues: a systematic review and meta‐analysis. Aging Cell. 2020;19(2):e13083. doi:10.1111/acel.13083 31808308 PMC6996941

[31] Koyama A , O’Brien J, Weuve J, Blacker D, Metti AL, Yaffe K. The role of peripheral inflammatory markers in dementia and Alzheimer’s disease: a meta-analysis. J Gerontol A Biol Sci Med Sci. 2013;68(4):433–440. doi:10.1093/gerona/gls187 22982688 PMC3693673

[32] Fulop T , Larbi A, Pawelec G, et al. Immunology of aging: the birth of inflammaging. Clin Rev Allergy Immunol. 2021:1–14. doi:10.1007/s12016-021-08899-6 PMC8449217 34536213

[33] Ramsey KA , Rojer AG, D’Andrea L, et al. The association of objectively measured physical activity and sedentary behavior with skeletal muscle strength and muscle power in older adults: a systematic review and meta-analysis. Ageing Res Rev. 2021;67:101266. doi:10.1016/j.arr.2021.101266 33607291

[34] Rezuş E , Burlui A, Cardoneanu A, et al. Inactivity and skeletal muscle metabolism: a vicious cycle in old age. Int J Mol Sci. 2020;21(2):592. doi:10.3390/ijms21020592 PMC7014434 31963330

[35] Akbari M , Hassan-Zadeh V. IL-6 signalling pathways and the development of type 2 diabetes. Inflammo Pharmacol. 2018;26(3):685–698. doi:10.1007/s10787-018-0458-0 29508109

[36] Sadeghabadi ZA , Abbasalipourkabir R, Mohseni R, Ziamajidi N. Investigation of oxidative stress markers and antioxidant enzymes activity in newly diagnosed type 2 diabetes patients and healthy subjects, association with IL-6 level. J Diabetes Metab Disord. 2019;18(2):437–443. doi:10.1007/s40200-019-00437-8 31890669 PMC6915251

[37] Darweesh SK , Wolters FJ, Ikram MA, de Wolf F, Bos D, Hofman A. Inflammatory markers and the risk of dementia and Alzheimer’s disease: a meta-analysis. Alzheimer’s Dement. 2018;14(11):1450–1459. doi:10.1016/j.jalz.2018.02.014 29605221

[38] Shen X-N , Niu L-D, Wang Y-J, et al. Inflammatory markers in Alzheimer’s disease and mild cognitive impairment: a meta-analysis and systematic review of 170 studies. J Neurol Neurosurg Psychiatry. 2019;90(5):590–598. doi:10.1136/jnnp-2018-319148 30630955

[39] Krabbe KS , Pedersen M, Bruunsgaard H. Inflammatory mediators in the elderly. Exp Gerontol. 2004;39(5):687–699. doi:10.1016/j.exger.2004.01.009 15130663

[40] Villeda SA , Luo J, Mosher KI, et al. The ageing systemic milieu negatively regulates neurogenesis and cognitive function. Nature. 2011;477(7362):90–94. doi:10.1038/nature10357 21886162 PMC3170097

[41] Ferreira ST , Clarke JR, Bomfim TR, De Felice FG. Inflammation, defective insulin signaling, and neuronal dysfunction in Alzheimer’s disease. Alzheimer’s Dement. 2014;10(1):S76–S83. doi:10.1016/j.jalz.2013.12.010 24529528

[42] Dilger RN , Johnson RW. Aging, microglial cell priming, and the discordant central inflammatory response to signals from the peripheral immune system. J Leukoc Biol. 2008;84(4):932–939. doi:10.1189/jlb.0208108 18495785 PMC2538600

[43] Maher F , Nolan Y, Lynch MA. Downregulation of IL-4-induced signalling in hippocampus contributes to deficits in LTP in the aged rat. Neurobiol Aging. 2005;26(5):717–728. doi:10.1016/j.neurobiolaging.2004.07.002 15708447

[44] Blasko I , Grubeck-Loebenstein B. Role of the immune system in the pathogenesis, prevention and treatment of Alzheimer’s disease. Drugs Aging. 2003;20(2):101–113. doi:10.2165/00002512-200320020-00002 12534311

[45] Pedersen BK , Febbraio M. Muscle-derived interleukin-6—a possible link between skeletal muscle, adipose tissue, liver, and brain. Brain Behav Immun. 2005;19(5):371–376. doi:10.1016/j.bbi.2005.04.008 15935612

[46] Schumertl T , Lokau J, Rose-John S, Garbers C. Function and proteolytic generation of the soluble interleukin-6 receptor in health and disease. Biochim Biophys Acta Mol Cell Res. 2022;1869(1):119143. doi:10.1016/j.bbamcr.2021.119143 34626681

[47] Rose-John S . Therapeutic targeting of IL-6 trans-signaling. Cytokine. 2021;144:155577. doi:10.1016/j.cyto.2021.155577 34022535

[48] Garbers C , Heink S, Korn T, Rose-John S. Interleukin-6: designing specific therapeutics for a complex cytokine. Nat Rev Drug Discovery. 2018;17(6):395–412. doi:10.1038/nrd.2018.45 29725131

[49] Burton MD , Sparkman NL, Johnson RW. Inhibition of interleukin-6 trans-signaling in the brain facilitates recovery from lipopolysaccharide-induced sickness behavior. J Neuroinflammation. 2011;8(1):1–13. doi:10.1186/1742-2094-8-54 21595956 PMC3113341

[50] Kraakman MJ , Kammoun HL, Allen TL, et al. Blocking IL-6 trans-signaling prevents high-fat diet-induced adipose tissue macrophage recruitment but does not improve insulin resistance. Cell Metab. 2015;21(3):403–416. doi:10.1016/j.cmet.2015.02.006 25738456

[51] Campbell IL , Erta M, Lim SL, et al. Trans-signaling is a dominant mechanism for the pathogenic actions of interleukin-6 in the brain. J Neurosci. 2014;34(7):2503–2513. doi:10.1523/JNEUROSCI.2830-13.2014 24523541 PMC6802757

[52] Pedersen BK . Muscles and their myokines. J Exp Biol. 2011 2011;214:337–346. doi:10.1242/jeb.048074 21177953

[53] Brandt C , Pedersen BK. The role of exercise-induced myokines in muscle homeostasis and the defense against chronic diseases. Biomed Res Int. 2010;2010:1–6. doi:10.1155/2010/520258 PMC2836182 20224659

[54] Matthews VB , Åström M-B, Chan M, et al. Brain-derived neurotrophic factor is produced by skeletal muscle cells in response to contraction and enhances fat oxidation via activation of AMP-activated protein kinase. Diabetologia. 2009;52(7):1409–1418. doi:10.1007/s00125-009-1364-1 19387610

[55] Pedersen BK . Exercise-induced myokines and their role in chronic diseases. Brain Behav Immun. 2011;25(5):811–816. doi:10.1016/j.bbi.2011.02.010 21354469

[56] Singh-Manoux A , Dugravot A, Brunner E, et al. Interleukin-6 and C-reactive protein as predictors of cognitive decline in late midlife. Neurology. 2014;83(6):486–493. doi:10.1212/WNL.0000000000000665 24991031 PMC4141998

[57] Komaki G , Arimura A, Koves K. Effect of intravenous injection of IL-1 beta on PGE2 levels in several brain areas as determined by microdialysis. Am J Physiol Endocrinol Metab. 1992;262(2):E246–E251. doi:10.1152/ajpendo.1992.262.2.e246 1539653

[58] Pedersen BK . Physical activity and muscle–brain crosstalk. Nat Rev Endocrinol. 2019;15(7):383–392. doi:10.1038/s41574-019-0174-x 30837717

[59] Moon HY , Becke A, Berron D, et al. Running-induced systemic cathepsin B secretion is associated with memory function. Cell Metab. 2016;24(2):332–340. doi:10.1016/j.cmet.2016.05.025 27345423 PMC6029441

[60] Komulainen P , Pedersen M, Hänninen T, et al. BDNF is a novel marker of cognitive function in ageing women: the DR’s EXTRA Study. Neurobiol Learn Mem. 2008;90(4):596–603. doi:10.1016/j.nlm.2008.07.014 18707012

[61] Krabbe KS , Mortensen EL, Avlund K, et al. Brain‐derived neurotrophic factor predicts mortality risk in older women. J Am Geriatr Soc. 2009;57(8):1447–1452. doi:10.1111/j.1532-5415.2009.02345.x 19515111

[62] Xie B , Zhou H, Liu W, et al. Evaluation of the diagnostic value of peripheral BDNF levels for Alzheimer’s disease and mild cognitive impairment: results of a meta-analysis. Int J Neurosci. 2020;130(3):218–230. doi:10.1080/00207454.2019.1667794 31518516

[63] Calabrese F , Rossetti AC, Racagni G, Gass P, Riva MA, Molteni R. Brain-derived neurotrophic factor: a bridge between inflammation and neuroplasticity. Front Cell Neurosci. 2014;8:430. doi:10.3389/fncel.2014.00430 25565964 PMC4273623

[64] Liu PZ , Nusslock R. Exercise-mediated neurogenesis in the hippocampus via BDNF. Front Neurosci. 2018;12:1–6. doi:10.3389/fnins.2018.00052 29467613 PMC5808288

[65] Lourenco MV , Frozza RL, de Freitas GB, et al. Exercise-linked FNDC5/irisin rescues synaptic plasticity and memory defects in Alzheimer’s models. Nat Med. 2019;25(1):165–175. doi:10.1038/s41591-018-0275-4 30617325 PMC6327967

[66] Lourenco MV , Ribeiro FC, Sudo FK, et al. Cerebrospinal fluid irisin correlates with amyloid‐β, BDNF, and cognition in Alzheimer’s disease. Alzheimer’s Dement. 2020;12(1):e12034. doi:10.1002/dad2.12034 PMC7306518 32582833

[67] Sylow L , Tokarz VL, Richter EA, Klip A. The many actions of insulin in skeletal muscle, the paramount tissue determining glycemia. Cell Metab. 2021;33(4):758–780. doi:10.1016/j.cmet.2021.03.020 33826918

[68] Kim K , Park SM. Association of muscle mass and fat mass with insulin resistance and the prevalence of metabolic syndrome in Korean adults: a cross-sectional study. Sci Rep. 2018;8(1):1–8. doi:10.1038/s41598-018-21168-5 29426839 PMC5807388

[69] Ekblad LL , Rinne JO, Puukka P, et al. Insulin resistance predicts cognitive decline: an 11-year follow-up of a nationally representative adult population sample. Diabetes Care. 2017;40(6):751–758. doi:10.2337/dc16-2001 28381479

[70] Chang AM , Halter JB. Aging and insulin secretion. Am J Physiol Endocrinol Metab. 2003;284(1):E7–E12. doi:10.1152/ajpendo.00366.2002 12485807

[71] Thambisetty M , Beason-Held LL, An Y, et al. Impaired glucose tolerance in midlife and longitudinal changes in brain function during aging. Neurobiol Aging. 2013;34(10):2271–2276. doi:10.1016/j.neurobiolaging.2013.03.025 23608110 PMC4577027

[72] Mergenthaler P , Lindauer U, Dienel GA, Meisel A. Sugar for the brain: the role of glucose in physiological and pathological brain function. Trends Neurosci. 2013;36(10):587–597. doi:10.1016/j.tins.2013.07.001 23968694 PMC3900881

[73] Roh E , Kim M-S. Emerging role of the brain in the homeostatic regulation of energy and glucose metabolism. Exp Mol Med. 2016;48(3):e216–e216. doi:10.1038/emm.2016.4 26964832 PMC4892882

[74] Cholerton B , Baker LD, Craft S. Insulin, cognition, and dementia. Eur J Pharmacol. 2013;719(1-3):170–179. doi:10.1016/j.ejphar.2013.08.008 24070815 PMC5405627

[75] Nguyen TT , Ta QTH, Nguyen TTD, Le TT. Role of insulin resistance in the Alzheimer’s Disease progression. Neurochem Res. 2020;45(7):1481–1491. doi:10.1007/s11064-020-03031-0 32314178

[76] Rhea EM , Banks WA. A historical perspective on the interactions of insulin at the blood‐brain barrier. J Neuroendocrinol. 2021;33(4):e12929. doi:10.1111/jne.12929 33433042 PMC8052275

[77] Barclay RD , Burd NA, Tyler C, Tillin NA, Mackenzie RW. The role of the IGF-1 signaling cascade in muscle protein synthesis and anabolic resistance in aging skeletal muscle. Front Nutr. 2019;6:146. doi:10.3389/fnut.2019.00146 31552262 PMC6746962

[78] Jarmusch S , Baber L, Bidlingmaier M, et al. Influence of IGF-I serum concentration on muscular regeneration capacity in patients with sarcopenia. BMC Musculoskelet Disord. 2021;22(1):1–11. doi:10.1186/s12891-021-04699-3 34544407 PMC8454138

[79] Bian A , Ma Y, Zhou X, et al. Association between sarcopenia and levels of growth hormone and insulin-like growth factor-1 in the elderly. BMC Musculoskelet Disord. 2020;21(1):1–9. doi:10.1186/s12891-020-03236-y PMC7140321 32264885

[80] Wennberg AM , Hagen CE, Machulda MM, et al. The association between peripheral total IGF-1, IGFBP-3, and IGF-1/IGFBP-3 and functional and cognitive outcomes in the Mayo Clinic Study of Aging. Neurobiol Aging. 2018;66:68–74. doi:10.1016/j.neurobiolaging.2017.11.017 29547749 PMC5924628

[81] Kim IY , Park S, Jang J, Wolfe RR. Understanding muscle protein dynamics: technical considerations for advancing sarcopenia research. Ann Geriatr Med Res. Sep 2020;24(3):157–165. doi:10.4235/agmr.20.0041 32752586 PMC7533194

[82] Burd NA , Gorissen SH, Van Loon LJ. Anabolic resistance of muscle protein synthesis with aging. Exerc Sport Sci Rev. 2013;41(3):169–173. doi:10.1097/JES.0b013e318292f3d5 23558692

[83] Anisimova AS , Alexandrov AI, Makarova NE, Gladyshev VN, Dmitriev SE. Protein synthesis and quality control in aging. Aging (Albany NY). 2018;10(12):4269. doi:10.18632/aging.101721 30562164 PMC6326689

[84] Götz J , Ittner LM. Animal models of Alzheimer’s disease and frontotemporal dementia. Nat Rev Neurosci. 2008;9(7):532–544. doi:10.1038/nrn2420 18568014

[85] Lovestone S , McLoughlin D. Protein aggregates and dementia: is there a common toxicity?. J Neurol Neurosurg Psychiatry. 2002;72(2):152–161. doi:10.1136/jnnp.72.2.152 11796764 PMC1737746

[86] Domínguez M , de Oliveira E, Odena MA, Portero M, Pamplona R, Ferrer I. Redox proteomic profiling of neuroketal-adducted proteins in human brain: regional vulnerability at middle age increases in the elderly. Free Radic Biol Med. 2016;95:1–15. doi:10.1016/j.freeradbiomed.2016.02.034 26968793

[87] Al Mamun A , Uddin MS, Kabir MT, et al. Exploring the promise of targeting ubiquitin-proteasome system to combat Alzheimer’s disease. Neurotox Res. 2020;38(1):8–17. doi:10.1007/s12640-020-00185-1 32157628

[88] Sakuma K , Yamaguchi A. Recent advances in pharmacological, hormonal, and nutritional intervention for sarcopenia. Pflugers Arch. 2018;470(3):449–460. doi:10.1007/s00424-017-2077-9 29043432

[89] Gan Z , Fu T, Kelly DP, Vega RB. Skeletal muscle mitochondrial remodeling in exercise and diseases. Cell Res. 2018;28(10):969–980. doi:10.1038/s41422-018-0078-7 30108290 PMC6170448

[90] Liguori I , Russo G, Curcio F, et al. Oxidative stress, aging, and diseases. Clin Interv Aging. 2018;13:757–772. doi:10.2147/CIA.S158513 29731617 PMC5927356

[91] Tobore TO . On the central role of mitochondria dysfunction and oxidative stress in Alzheimer’s disease. Neurol Sci. 2019;40(8):1–14. doi:10.1007/s10072-019-03863-x 30982132

[92] Khacho M , Harris R, Slack RS. Mitochondria as central regulators of neural stem cell fate and cognitive function. Nat Rev Neurosci. 2019;20(1):34–48. doi:10.1038/s41583-018-0091-3 30464208

[93] McKenzie D , Bua E, McKiernan S, Cao Z, Wanagat J, Aiken JM. Mitochondrial DNA deletion mutations: a causal role in sarcopenia. Eur J Biochem. 2002;269(8):2010–2015. doi:10.1046/j.1432-1033.2002.02867.x 11985577

[94] Marzetti E , Calvani R, Cesari M, et al. Mitochondrial dysfunction and sarcopenia of aging: from signaling pathways to clinical trials. Int J Biochem cell Biol. 2013;45(10):2288–2301. doi:10.1016/j.biocel.2013.06.024 23845738 PMC3759621

[95] Chapman J , Fielder E, Passos JF. Mitochondrial dysfunction and cell senescence: deciphering a complex relationship. FEBS Lett. 2019;593(13):1566–1579. doi:10.1002/1873-3468.13498 31211858

[96] White AR , Du T, Laughton KM, et al. Degradation of the Alzheimer disease amyloid β-peptide by metal-dependent up-regulation of metalloprotease activity. J Biol Chem. 2006;281(26):17670–17680. doi:10.1074/jbc.M602487200 16648635

[97] Limoge M , Safina A, Beattie A, Kapus L, Truskinovsky AM, Bakin AV. Tumor-fibroblast interactions stimulate tumor vascularization by enhancing cytokine-driven production of MMP9 by tumor cells. Oncotarget. 2017;8(22):35592–35608. doi:10.18632/oncotarget.16022 28423685 PMC5482601

[98] Rocha M , Hernandez-Mijares A, Garcia-Malpartida K, Banuls C, Bellod L, Victor V M. Mitochondria-targeted antioxidant peptides. Curr Pharm Des. 2010;16(28):3124–3131. doi:10.2174/138161210793292519 20687871

[99] Turrens JF . Mitochondrial formation of reactive oxygen species. J Physiol. 2003;552(2):335–344. doi:10.1113/jphysiol.2003.049478 14561818 PMC2343396

[100] Leuner K , Schütt T, Kurz C, et al. Mitochondrion-derived reactive oxygen species lead to enhanced amyloid beta formation. Antioxid Redox Signal. 2012;16(12):1421–1433. doi:10.1089/ars.2011.4173 22229260 PMC3329950

[101] Wong KY , Roy J, Fung ML, Heng BC, Zhang C, Lim LW. Relationships between mitochondrial dysfunction and neurotransmission failure in Alzheimer’s disease. Aging Dis. 2020;11(5):1291–1316. doi:10.14336/AD.2019.1125 33014538 PMC7505271

[102] Swerdlow RH . Mitochondria and mitochondrial cascades in Alzheimer’s disease. J Alzheimers Dis. 2018;62(3):1403–1416. doi:10.3233/JAD-170585 29036828 PMC5869994

[103] Swerdlow RH , Burns JM, Khan SM. The Alzheimer’s disease mitochondrial cascade hypothesis: progress and perspectives. Biochim Biophys Acta Mol Basis Dis. 2014;1842(8):1219–1231. doi:10.1016/j.bbadis.2013.09.010 PMC3962811 24071439

[104] Ismail R , Parbo P, Madsen LS, et al. The relationships between neuroinflammation, beta-amyloid and tau deposition in Alzheimer’s disease: a longitudinal PET study. J Neuroinflammation. 2020;17:1–11. doi:10.1186/s12974-020-01820-6 32375809 PMC7203856

[105] Wrann CD , White JP, Salogiannnis J, et al. Exercise induces hippocampal BDNF through a PGC-1α/FNDC5 pathway. Cell Metab. 2013;18(5):649–659. doi:10.1016/j.cmet.2013.09.008 24120943 PMC3980968

[106] Funk JA , Gohlke J, Kraft AD, McPherson CA, Collins JB, Harry GJ. Voluntary exercise protects hippocampal neurons from trimethyltin injury: possible role of interleukin-6 to modulate tumor necrosis factor receptor-mediated neurotoxicity. Brain Behav Immun. 2011;25(6):1063–1077. doi:10.1016/j.bbi.2011.03.012 21435392 PMC3138904

[107] Burtscher J , Millet GP, Place N, Kayser B, Zanou N. The muscle-brain axis and neurodegenerative diseases: the key role of mitochondria in exercise-induced neuroprotection. Int J Mol Sci. 2021;22(12):6479. doi:10.3390/ijms22126479 34204228 PMC8235687

[108] Cheng A , Wan R, Yang J-L, et al. Involvement of PGC-1α in the formation and maintenance of neuronal dendritic spines. Nat Commun. 2012;3(1):1–12. doi:10.1038/ncomms2238 PMC4091730 23212379

[109] Eisele PS , Salatino S, Sobek J, Hottiger MO, Handschin C. The peroxisome proliferator-activated receptor γ coactivator 1α/β (PGC-1) coactivators repress the transcriptional activity of NF-κB in skeletal muscle cells. J Biol Chem. 2013;288(4):2246–2260. doi:10.1074/jbc.M112.375253 23223635 PMC3554897

[110] Chwieralski C , Welte T, Bühling F. Cathepsin-regulated apoptosis. Apoptosis. 2006;11(2):143–149. doi:10.1007/s10495-006-3486-y 16502253

[111] Allen MD , Dalton BH, Gilmore KJ, et al. Neuroprotective effects of exercise on the aging human neuromuscular system. Exp Gerontol. 2021;152:111465. doi:10.1016/j.exger.2021.111465 34224847

[112] Correia JC , Kelahmetoglu Y, Jannig PR, et al. Muscle-secreted neurturin couples myofiber oxidative metabolism and slow motor neuron identity. Cell Metab. 2021;33(11):2215–2230.e8. doi:10.1016/j.cmet.2021.09.003 34592133

[113] Van Dam R , Van Ancum JM, Verlaan S, Scheerman K, Meskers CG, Maier AB. Lower cognitive function in older patients with lower muscle strength and muscle mass. Dement Geriatr Cogn Disord. 2018;45:243–250. doi:10.1159/000486711 29913450 PMC6067649

[114] Taekema DG , Ling CH, Kurrle SE, et al. Temporal relationship between handgrip strength and cognitive performance in oldest old people. Age Ageing. 2012;41(4):506–512. doi:10.1093/ageing/afs013 22374646

[115] Ogawa Y , Kaneko Y, Sato T, Shimizu S, Kanetaka H, Hanyu H. Sarcopenia and muscle functions at various stages of Alzheimer disease. Front Neurol. 2018;9:1–7. doi:10.3389/fneur.2018.00710 30210435 PMC6121095

[116] Du Z , Li Y, Li J, Zhou C, Li F, Yang X. Physical activity can improve cognition in patients with Alzheimer’s disease: a systematic review and meta-analysis of randomized controlled trials. Clin Interv Aging. 2018;13:1593–1603. doi:10.2147/CIA.S169565 30233156 PMC6130261

[117] Karssemeijer EE , Aaronson JJ, Bossers WW, Smits TT, Kessels RR. Positive effects of combined cognitive and physical exercise training on cognitive function in older adults with mild cognitive impairment or dementia: a meta-analysis. Ageing Res Rev. 2017;40:75–83. doi:10.1016/j.arr.2017.09.003 28912076

[118] Côté S , Carmichael P-H, Verreault R, Lindsay J, Lefebvre J, Laurin D. Nonsteroidal anti-inflammatory drug use and the risk of cognitive impairment and Alzheimer’s disease. Alzheimers Dement. 2012;8(3):219–226. doi:10.1016/j.jalz.2011.03.012 22546354

[119] Szekely CA , Thorne JE, Zandi PP, et al. Nonsteroidal anti-inflammatory drugs for the prevention of Alzheimer’s disease: a systematic review. Neuroepidemiology. 2004;23(4):159–169. doi:10.1159/000078501 15279021

[120] de Freitas GB , Lourenco MV, De Felice FG. Protective actions of exercise‐related FNDC5/Irisin in memory and Alzheimer’s disease. J Neurochem. 2020;155(6):602–611. doi:10.1111/jnc.15039 32396989

