# Supplementary material for: Pathophysiological Mechanisms Explaining the Association Between Low Skeletal Muscle Mass and Cognitive Function
Source: J Gerontol A Biol Sci Med Sci. 2022 Jun 6;77(10):1959–68. doi: 10.1093/gerona/glac121 (PMC9536455; doi:10.1093/gerona/glac121)
Supplement: glac121_suppl_Supplementary_Appendix [file glac121_suppl_supplementary_appendix.docx]

**Appendix**

*Search strategy Embase*

'dementia'/de OR 'Alzheimer disease'/exp OR 'cognitive defect'/de OR 'mild cognitive impairment'/exp OR 'cognition'/de OR dementia:ti,ab,kw OR Alzheimer*:ti,ab,kw OR cogniti*:ti,ab,kw OR 'Neurocognitive Disorder*':ti,ab,kw

'muscle strength'/exp OR 'dynamometer'/de OR 'muscle atrophy'/exp OR 'body composition'/de OR 'muscle weakness'/de OR ‘muscle strength’:ti,ab,kw OR ‘muscular strength’:ti,ab,kw OR ‘muscle weak*’:ti,ab,kw OR ‘muscular weak*’:ti,ab,kw OR ‘muscle deter*’:ti,ab,kw OR ‘muscular deter*’:ti,ab,kw OR ‘sarcopeni*’:ti,ab,kw OR ‘grip strength’:ti,ab,kw OR ‘hand strength’:ti,ab,kw OR ‘quadriceps strength’:ti,ab,kw OR ‘dynapenia’:ti,ab,kw OR ‘dynamomet*’:ti,ab,kw OR ‘handgrip’:ti,ab,kw OR ‘hand grip’:ti,ab,kw OR ‘knee extension torque’:ti,ab,kw OR ‘body composition’:ti,ab,kw OR ‘lean mass’:ti,ab,kw OR ‘lean body mass’:ti,ab,kw OR ‘appendicular mass’:ti,ab,kw OR ‘fat free mass’:ti,ab,kw OR ‘FFM’:ti,ab,kw OR ‘ALM’:ti,ab,kw OR ‘fat free body mass’:ti,ab,kw OR ‘skeletal mass’:ti,ab,kw OR ‘SMI’:ti,ab,kw OR ‘muscle mass’:ti,ab,kw OR ‘muscle declin*’:ti,ab,kw OR ‘muscle decreas*’:ti,ab,kw OR ‘muscle atroph*’:ti,ab,kw OR ‘muscle wasting’:ti,ab,kw OR ‘muscle loss’:ti,ab,kw OR ‘loss of muscle’:ti,ab,kw OR ‘muscular mass’:ti,ab,kw OR ‘muscular declin*’:ti,ab,kw OR ‘muscular decreas*’:ti,ab,kw OR ‘muscular atroph*’:ti,ab,kw OR ‘muscular wasting’:ti,ab,kw OR ‘muscular loss’:ti,ab,kw OR ‘cachexi*’:ti,ab,kw

'insulin resistance'/exp OR 'somatomedin c'/exp OR ‘Insulin Resistan*’:ti,ab,kw OR ‘Insulin-Like Growth Factor*’:ti,ab,kw

'inflammation'/de OR 'systemic inflammation'/exp OR 'systemic inflammatory response'/exp OR 'myokine'/exp OR 'systemic inflammat*':ti,ab,kw OR myokine*:ti,ab,kw

'serum albumin'/exp OR 'hypoproteinemia'/exp OR 'protein degradation'/exp OR ‘serum albumin*’:ti,ab,kw OR ‘plasma albumin*’:ti,ab,kw OR ‘hypoproteinemia’:ti,ab,kw OR ‘hypoalbuminemia’:ti,ab,kw OR ‘hypoproteinaemia’:ti,ab,kw OR ‘hypoalbuminaemia’:ti,ab,kw OR ‘proteolysis’:ti,ab,kw OR ‘proteolyses’:ti,ab,kw OR ‘protein degradation*’:ti,ab,kw OR ‘protein turnover’:ti,ab,kw

'mitochondrion'/exp OR 'mitochondrial dynamics'/exp OR 'mitophagy'/exp OR 'reactive oxygen metabolite'/exp OR 'oxidative stress'/exp OR ‘mitochondri*’:ti,ab,kw OR ‘ reactive oxygen species’:ti,ab,kw OR ‘oxygen radical*’:ti,ab,kw OR ‘free radical*’:ti,ab,kw OR ‘oxidative stress*’:ti,ab,kw

*Search strategy PubMed*

"Dementia"[Mesh:NoExp] OR "Alzheimer Disease"[Mesh] OR "Cognitive Dysfunction"[Mesh] OR "Cognition"[Mesh:NoExp] OR "Cognition Disorders"[Mesh:NoExp] OR dementia[tiab] OR Alzheimer*[tiab] OR cogniti*[tiab] OR Neurocognitive Disorder*[tiab]

"Muscle Strength"[Mesh] OR "Muscle Strength Dynamometer"[Mesh] OR "Muscular Atrophy"[Mesh] OR "Body Composition"[Mesh:noexp] OR "Muscle Weakness" [Mesh] OR muscle strength[tiab] OR muscular strength[tiab] OR muscle weak*[tiab] OR muscular weak*[tiab] OR muscle deter*[tiab] OR muscular deter*[tiab] OR sarcopeni*[tiab] OR grip strength[tiab] OR hand strength[tiab] OR quadriceps strength[tiab] OR dynapenia[tiab] OR dynamomet*[tiab] OR handgrip[tiab] OR hand grip[tiab] OR knee extension torque[tiab] OR body composition[tiab] OR lean mass[tiab] OR lean body mass[tiab] OR appendicular mass[tiab] OR fat free mass[tiab] OR FFM[tiab] OR ALM[tiab] OR fat free body mass[tiab] OR skeletal mass[tiab] OR SMI[tiab] OR muscle mass[tiab] OR muscle declin*[tiab] OR muscle decreas*[tiab] OR muscle atroph*[tiab] OR muscle wasting[tiab] OR muscle loss[tiab] OR loss of muscle[tiab] OR muscular mass[tiab] OR muscular declin*[tiab] OR muscular decreas*[tiab] OR muscular atroph*[tiab] OR muscular wasting[tiab] OR muscular loss[tiab] OR cachexi* [tiab]

"Insulin Resistance"[Mesh] OR "Insulin-Like Growth Factor I"[Mesh] OR Insulin Resistan*[tiab] OR Insulin-Like Growth Factor*[tiab]

"Inflammation"[Mesh] OR systemic inflammat*[tiab] OR myokine*[tiab]

"Serum Albumin"[Mesh] OR "Hypoproteinemia"[Mesh] OR "Proteolysis"[Mesh] OR serum albumin*[tiab] OR plasma albumin*[tiab] OR hypoproteinemia[tiab] OR hypoalbuminemia[tiab] OR hypoproteinaemia[tiab] OR hypoalbuminaemia[tiab] OR proteolysis[tiab] OR proteolyses[tiab] OR protein degradation*[tiab] OR protein turnover[tiab]

"Mitochondria"[Mesh] OR "Mitochondrial Turnover"[Mesh] OR "Reactive Oxygen Species"[Mesh:NoExp] OR "Oxidative Stress"[Mesh:NoExp] OR mitochondri*[tiab] OR reactive oxygen species[tiab] OR oxygen radical*[tiab] OR free radical*[tiab] OR oxidative stress*[tiab]
